# A novel 2D silicon nano-mold fabrication technique for linear nanochannels over a 4 inch diameter substrate

**DOI:** 10.1038/srep18921

**Published:** 2016-01-11

**Authors:** Zhifu Yin, Liping Qi, Helin Zou, Lei Sun

**Affiliations:** 1 Key Laboratory for Micro/Nano Technology and Systems of Liaoning Province, Dalian University of Technology, Dalian 116024, China; 2 Department of Biomedical Engineering, Dalian University of Technology, Dalian 116024, China; 3 Key Laboratory for Precision and Non-traditional Machining Technology of Ministry of Education, Dalian University of Technology, Dalian 116024, China

## Abstract

A novel low-cost 2D silicon nano-mold fabrication technique was developed based on Cu inclined-deposition and Ar^+^ (argon ion) etching. With this technique, sub-100 nm 2D (two dimensional) nano-channels can be etched economically over the whole area of a 4 inch n-type <100> silicon wafer. The fabricating process consists of only 4 steps, UV (Ultraviolet) lithography, inclined Cu deposition, Ar^+^ sputter etching, and photoresist & Cu removing. During this nano-mold fabrication process, we investigated the influence of the deposition angle on the width of the nano-channels and the effect of Ar^+^ etching time on their depth. Post-etching measurements showed the accuracy of the nanochannels over the whole area: the variation in width is 10%, in depth it is 11%. However, post-etching measurements also showed the accuracy of the nanochannels between chips: the variation in width is 2%, in depth it is 5%. With this newly developed technology, low-cost and large scale 2D nano-molds can be fabricated, which allows commercial manufacturing of nano-components over large areas.

Nanofluidics is the analysis of the behavior, manipulation, and control of fluids when they are confined to nanometer characteristic dimensions^1^. For the past five years, many new research methods and tools have been developed in this scientific discipline^2^,^3^ making use of various newly explored properties of nanochannels, including their enormously high surface-to-volume ratio that leads to capillarity-induced negative pressure in fluids they contain^4^,^5^; overlapping electrical double layers caused by the electrostatic interaction between the charged nanochannel surface and fluid ions, allowing ion transport controlled by surface charge^6^,^7^; and the dimensional proximity between nanochannels and biomolecules that facilitates molecule selectivity and stretching^3^,^8^. Already throughout the past decade, nanofluidic devices have become ubiquitous tools for the study of nano-scale fluidic, molecular and ion properties. Thanks to all these newly discovered transport or selectivity phenomena, nanofluidics has already found widespread applications in many biological and chemical fields, including protein analysis^9^,^10^, DNA stretching and mapping^11^,^12^,^13^, virus characterization^14^ and ion separation^15^,^16^. This field is even now expanding to address the critical challenges the macro world is facing. For example, new solutions for energy absorption^17^,^18^ and water purification^19^,^20^ have been proposed based on nanofluidic principles. There is thus much to be gained from developing fabrication methods that allow a simple, timely and low-cost production of reliable, accurate nanofluidic devices.

With the development of nanofluidics, large-scale nanochannel fabrication has been gaining in significance, offering exciting opportunities for both fundamental research and innovative applications^21^. Future advances in nanofluidics and its utilization in commercial applications depends on the development and fabrication of low cost and high fidelity nano-mold. In this study we are presenting a novel way of fabricating large-surface nano-molds (4 inches in diameter) that are to be used in the high-volume production of nanochannel devices. One of the challenges has been to assure a uniform width and depth of the nanochannels over such a large area without making the whole process too time-consuming and too costly.

Several other methods have been tried so far. They can be grouped as follows:

1. Beam-based methods, where a proton beam (PB)^8^,^22^, electron beam (EB)^23^,^24^,^25^, or focused ion beam (FIB)^26^,^27^ is used to fabricate 2D nanochannels on silicon or quartz. These procedures are not complicated but very costly and time-consuming;

2. Slightly more economical special methods that are more complicated and require costly equipment: chemical-mechanical polishing^28^, sacrificial layer surface-machining^29^, interference lithography^30^,^31^, and sidewall transfer techniques^32^,^33^,^34^. However, with these methods, the fabrication process is difficult to control, and the resulting 2D nanochannels over a large area lack uniformity. Furthermore, other procedures that drive up costs may need to be added, such as enhanced chemical vapor deposition (PECVD), radio frequency (RF) gold sputtering, interferometric lithography, reactive ion etching (RIE) or deep reactive ion etching (DRIE).

The method proposed in this study has several advantages over previous ones: (a) it follows four simple steps, as shown in Fig. 1 (UV-photolithography, inclined Cu deposition, Ar^+^ sputter etching, photoresist & Cu removal); (b) it is economical; and (c) high reproducibility fabrication. What needed to be determined in this study were the reliably uniform dimensions of different size nanochannels over the entire large area substrate as well as equally uniform nanochannels in the fabricated end products, the nanochannel devices.

## Experiments

**Figure 1:**
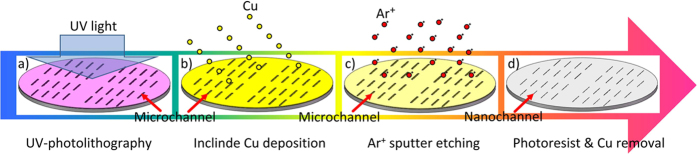
The diagram of 4 fabrication process. (**a**) transfer of the microchannels by UV-photolithography on the substrate spin-coated with AZ703 photoresist; (**b**) application by inclined Cu deposition of a 200 nm Cu nano-mask over the substrate with its grate of photoresist mesas; (**c**) Ar^+^ etching of nano-mask along the lines where the silicon substrate was not covered by the Cu layer; (**d**) removal of the Cu layer and photoresist mesas by cleaning with acid.

The 4 inch 2D nanochannel mold was fabricated in 4 steps, which consisted of UV-photolithography, inclined Cu deposition, Ar^+^ sputter etching, and photoresist & Cu removal, as shown in Fig. 1.

### Fabrication of a 2D 4 inch silicon nanochannel mold

**Figure 2:**
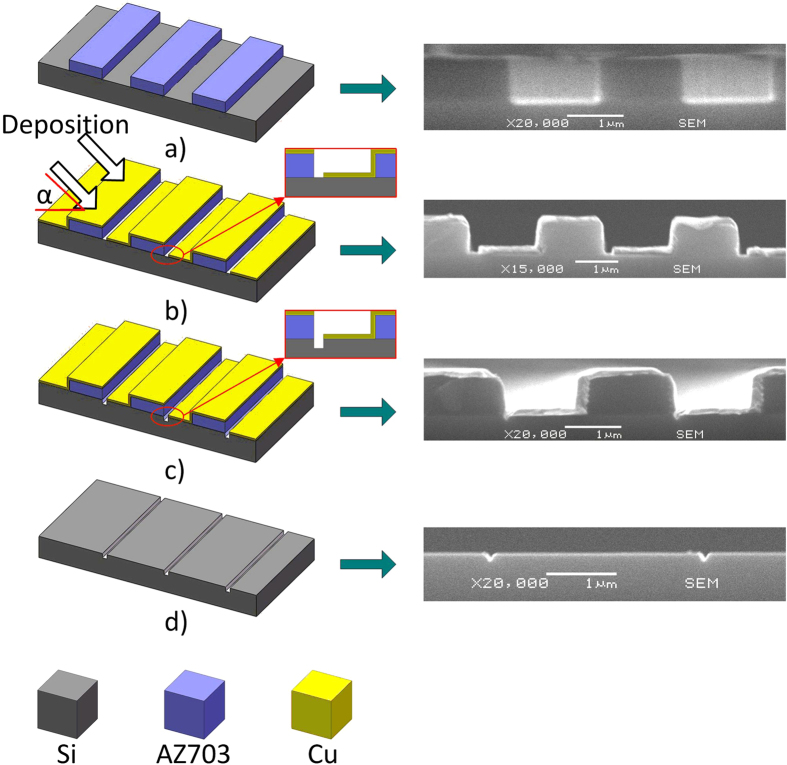
Illustration of the 2D 4 inch silicon nanochannels fabrication process. The images at right side show the SEM (scanning electron microscope) profiles of the silicon substrate during the 4 steps. (**a**) UV-photolithography, (**b**) inclined Cu deposition, (**c**) Ar^+^ sputter etching and (**d**) photoresist & Cu removal. α is the Cu deposition angle.

Figure 2 illustrates the 2D 4 inch silicon nanochannel fabrication process based on inclined Cu deposition and Ar^+^ etching. Figure 2a, UV-photolithography: Fabrication started with a 4 inch n-type <100> silicon wafer about 500 μm in thickness. The silicon substrate was cleaned with H_2_SO_4_, HF (hydrofluoric acid) and de-ionized water, and dried at 150 °C for 30 min. The heated substrate wafer was pre-treated for 20 min with hexamethyldisilazane (HMDS) from the gaseous phase to promote adhesion of the photoresist on its surface. A layer of positive photoresist (AZ703, Shipley Company, Massachusetts, US), approximately 400 nm thick, was spin coated on the substrate at a speed of 7000 rpm for 30 s. To avoid re-adsorption of water vapor, the coating operation was performed directly after cooling down the silicon substrate. Then with a mask consisting of linear grating pattern, 1.5 μm wide, 4 mm long, the photoresist was exposed to UV light for 21 s by a conventional contact mask aligner (MA/BA6, SUSS MicroTec, Germany). After developing the substrate for 30 s, the pattern remained as photoresist mesas on the substrate (the SEM image in Fig. 2a). The grating pattern covers the whole 4 inch silicon substrate.

Figure 2b, inclined Cu deposition: Inclined Cu deposition is an effective method for rapid fabrication of template layers. Cu, a relatively inexpensive deposition material, further reduces the fabrication cost. Before Cu deposition, the wafer was cleaned in oxygen plasma for 1 min to remove any residues in the channels. Then inclined Cu deposition was performed in a metal film thermal deposition system (ZZS400, costar group, China). The chamber pressure was maintained at 0.7 × 10^−3 ^Pa, with a working current of 155 A. During deposition, Cu source is melted under high working current and then the Cu is gasified and diffused to the substrate. Because of the anisotropy of the Cu deposition, the Cu can diffuse to the substrate at an angle, as shown in Fig. 2b. By precisely controlling the deposition angle, the Cu nano-mask can be fabricated (the SEM image in Fig. 2b).

Figure 2c, Ar^+^ sputter etching: Ar^+^ sputter etching was performed in a thin film deposition system (Kurt J. Lesker LAB18, France). In the process of sputter etching, the shutter plate is used as the positive electrode and the wafer holder plate is used as the negative electrode. The distance between the two plates was set to be 36 mm. A parallel plate electric field generated between these two plates, where argon ions vertically bombard the wafer. Therefore, the silicon without the protection of the Cu nano-mask is etched, as shown in the enlarged view and SEM image in Fig. 2c. The parameters of Ar^+^ sputter etching in this process were as follows: chamber power of 100 W, Ar pressure of 5 mtorr, and etching time of 30 min.

Figure 2d, photoresist & Cu removal: firstly, the substrate was immersed in a boiling Hcl and H_2_O_2_ solution (v:v, 1:3) for 5 min, then immersed in HF solution for 5 min to remove the oxidation layer generated in the Ar^+^ sputter etching process. Secondly, the silicon substrate was immersed in the boiled aqua regia (Hcl:HNO_3_ = 3:1) to remove the residual Cu and photoresist on the silicon substrate. Finally, the substrate was cleaned by a No.1 lotion (water: H_2_O_2_: ammonia water = 5:2:1, v/v) and a No.2 lotion (water:H_2_O_2_:Hcl = 8:2:1, v/v) to remove the impurities on the surface of the silicon substrate.

### Statistical Analysis

Statistical analysis was performed using SPSS (SPSS 16, SPSS, Inc., Chicago, IL, USA). The Kolmogorov-Smirnov test (K-S) and Shapiro-Wilk (S-W) test were performed first to confirm data normality. Regression analysis was conducted to examine the relationship between Cu deposition angle and width of the Cu nano-mask. Repeated measures ANOVA (Analysis of Variance) was used to compare the width and depth measured in different chips to verify the reproducibility of the proposed technique. The level of significance adopted in the study was p < 0.05.

## Results and Discussion

### Uniformity of the micro-mesas over the 4 inch diameter area

**Figure 3:**
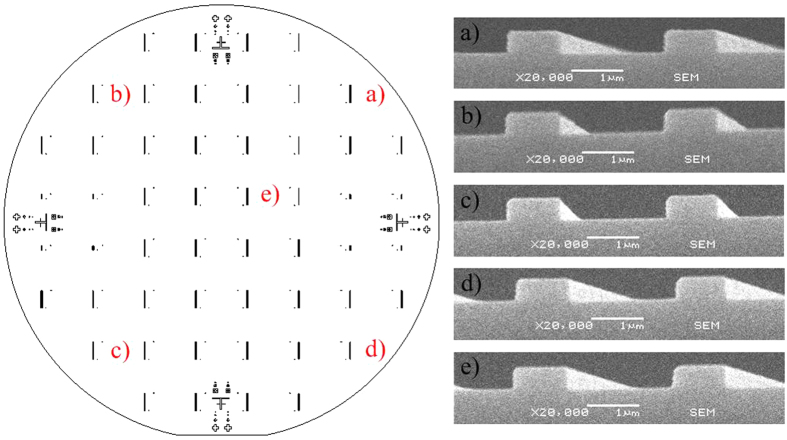
Photoresist micro-mesas in five different locations, (a) to (e), on a 4 inch diameter silicon wafer. The image on the left shows the locations chosen on the disk for measuring the height and width of the micro-mesas. The images on the right show the SEM images of these micro-mesas.

Nanochannel dimensional uniformity must be affected considerably by any tolerances occurring in the UV-photolithography step. It was thus necessary to investigate the uniformity of the resulting micro-mesas, which was done by measuring their height and width at five different locations. The image on the left of Fig. 3 shows the five locations on the substrate, (a) to (e), where mesas were chosen to be measured. After measuring three micro-mesas randomly selected at each location the average as well as standard deviation of height and width of the three were calculated. SEM images of these micro-mesas at locations (a) to (e) are shown on the right. Table 1 shows the average height and width of the micro-mesas at locations (a) to (e).

**Figure 4:**
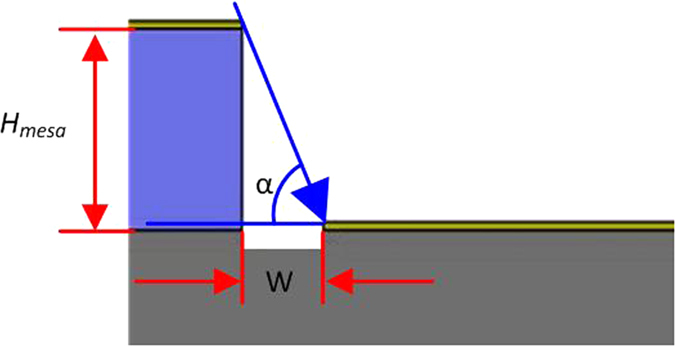
Influence of micro-mesa height and the Cu deposition angle on the width of the nanochannels.

Average height is 416 ± 3 nm, average width is 977 ± 25 nm. The maximum height difference among the micro-mesas is 8 nm, maximum width difference is 59 nm. Figure 2b illustrates the fact that micro-mesa width only affects the location and not the dimensions of the resulting nanochannel. Any difference in micro-mesa width can thus be ignored. Micro-mesa height, on the other hand, does affect the width of the nanochannels. The width difference of the nanochannels can be related to the height difference of the micro-mesa. That relation is illustrated in Fig. 4 and can be expressed in Eq. (1).


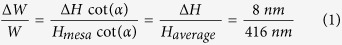


where, 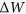 is the width difference of the nanochannel, *W* the width of the nanochannel,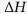 is the height difference of the micro-mesa, *H*_*mesa*_ the height of the micro-mesa and *α* the Cu deposition angle.

Variations in width of the resulting nanochannels attributable to the UV-photolithography process thus amount to less than 2% and can be ignored.

### The relationship between Cu deposition angle and width of the nanochannels

**Figure 5:**
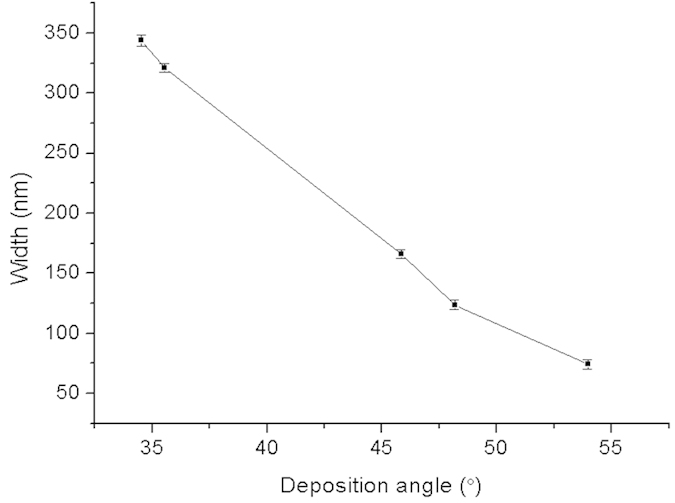
The relationship between Cu deposition angle and the width of the Cu nano-mask slits. The width of the Cu nano-mask slits decreases with a greater deposition angle.

The width of the resulting nanochannels is also influenced by the Cu nano-mask (Fig. 2b), as the width of its slits is determined by the Cu deposition angle. That width can be predicted by trigonometry, a steeper deposition angle being associated with narrower slits in the mask. Figure 5 shows the relationship between the Cu deposition angle and the width of the Cu nano-mask slits that follows a simple linear regression based on the Cu deposition angle. A significant regression equation was found (F (1, 13) = 992.65), *P* < 0.001), with a *R*^*2*^ = 0.987. The width of the Cu nano-mask slits could be predicted from the Cu deposition angle by the formula: *W* = 825.03 − 14.20 × *α,* where *W* is the width of the Cu nano-mask and *α* is the Cu deposition angle. The linear trend can be explained by means of trigonometry, see Supplementary Information SI for details.

When the Cu deposition angle is greater than 60°, the Cu completely covers the silicon substrate, and the width of the Cu nano-mask slits becomes 0.

### The relation between Ar^+^ sputter etching time and depth of the nanochannels

**Figure 6:**
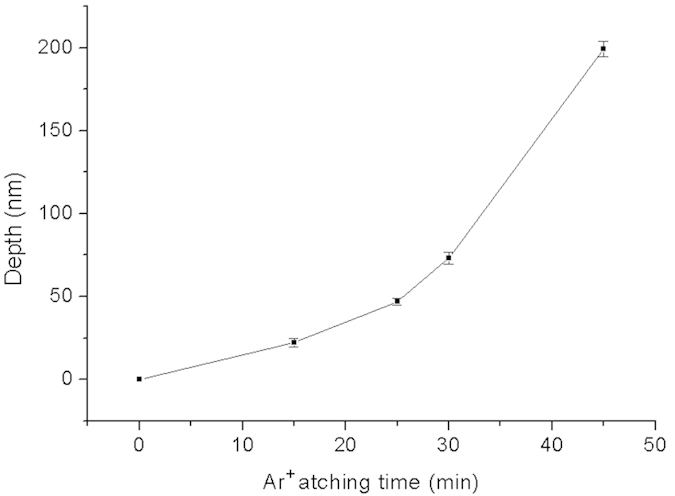
Relation between Ar^+^ etching time and depth of the nanochannels at a Cu deposition angle of 46.2°.

During the Ar^+^ sputter etching process, chamber power and Ar^+^ pressure were optimized at 100 W and 5 mtorr^35^. The depth of the resulting nanochannels is thus only affected by the Ar^+^ etching time (Fig. 2c). Figure 6 shows the relation between Ar^+^ etching time and the depth of the nanochannels, and the etching rate rises significantly with a longer Ar^+^ etching time. When the etching time is below 15 min, that rate remains at about 1.3 nm/min. But when the etching time is above 30 min, the etching rate increases to 7 nm/min. That increase stems from a higher sputtering rate related to the gradually rising substrate temperature after prolonged bombardment with argon ions. At the start of the etching process, the temperature of the substrate was 20.9 °C, rising to 46.4 °C after 30 min of continuous etching. At a higher substrate temperature, the sputter etching rate is higher^36^,^37^. For a better control of the etching rate, an etching-and-cooling process was proposed, see Supplementary Information SII for details.

### Uniformity of the 2D nanochannels over the 4 inch diameter area

**Figure 7:**
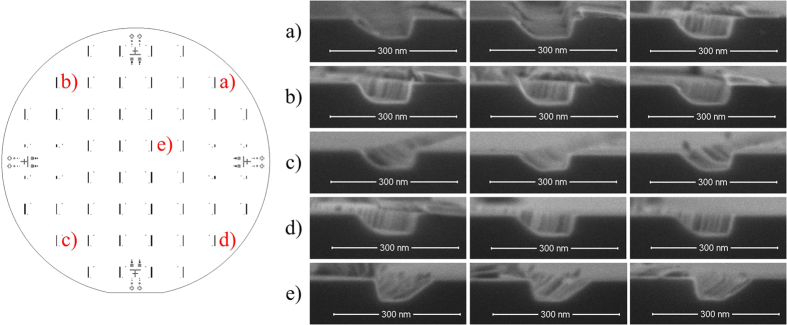
Five different nanochannel positions, (a) to (e), on the 4 inch diameter substrate. The image on the left shows the locations (**a**) to (**e**) on the disk chosen for measuring the width and depth of the nanochannels. The images on the right show the respective SEM images of the nanochannels. The Cu deposition angle was 46.2°, sputter etching time was 25 min.

To evaluate the uniformity of the nanochannels over the whole 4 inch diameter substrate, their width and depth was measured at five different locations on the disk. The image on the left in Fig. 7 shows their locations, (a) to (e); the corresponding SEM images are shown on the right. The Cu deposition angle was 46.2°, sputter etching time was 25 min.

After measuring the three randomly selected nanochannels at each location, the average width and depth of these three nanochannels was calculated, as well as the respective standard deviation.

**Figure 8:**
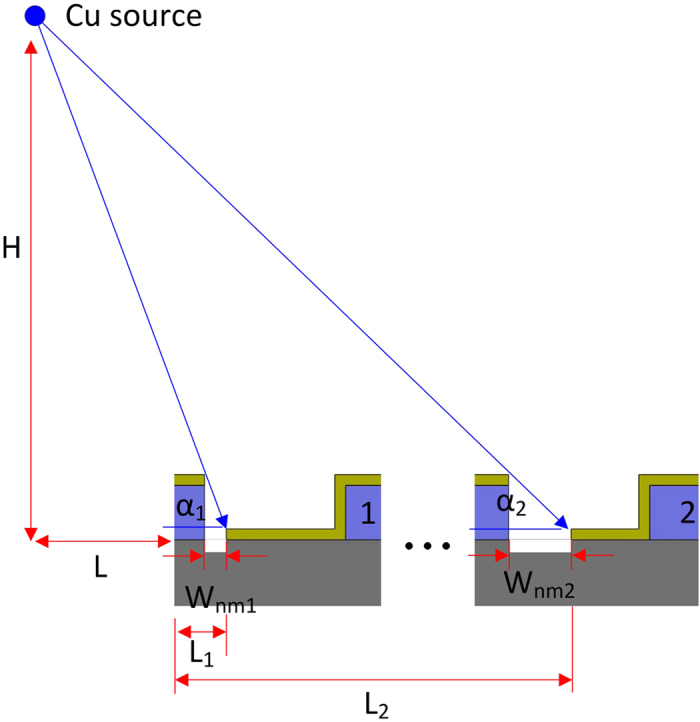
Width difference of the nanochannels over a 4 inch disk. *W*_*nm*_ is the width of the nano-mask slits, *α*_*1*_, *α*_*2*_ are the deposition angles, *H* is the vertical distance between Cu source and the covered substrate, *L* is the horizontal distance between the Cu source and each micro-mesa, *L*_*1*_ and *L*_*2*_ are the edge distances between the photoresist mesa and the Cu cover on the substrate.

Table 2 shows the average dimensions of the nanochannels and respective standard deviation at locations (a) to (e). The nanochannels at locations (b) and (c) are clearly, if minimally, less wide than those at locations (a) and (d). As shown in Fig. 8, that difference was caused by the slight difference of shadow widths (W_nm1_ < W_nm2_), during the Cu dengle *α*_*1*_, *α*_*2*_ can be expressed as


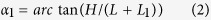



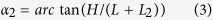


where, *α*_*1*_, *α*_*2*_ are the deposition angles, *H* is the vertical distance between the Cu source and the substrate, *L* is the horizontal distance between Cu source and the substrate, *L*_*1*_ and *L*_*2*_ are the edge distances between the photoresist mesa and the Cu cover on the substrate.

Because vertical distance *H* and horizontal distance *L* of the Cu source from the substrate are much greater than the edge distances *L*_*1*_ and *L*_*2*_, and *H*/(*L* + *L*_*1*_) equals approximately *H*/(*L* + *L*_*2*_), the deposition angle *α*_*1*_ equals approximately deposition angle *α*_*2*_. As shown in Table 2, the maximum difference in nanochannel width over the entire area is about 14 nm. The average width of the nanochannels on the wafer being 138 nm, this amounts to about 10% variation, a high enough value to be seen as a limitation of the proposed technique.

**Figure 9:**
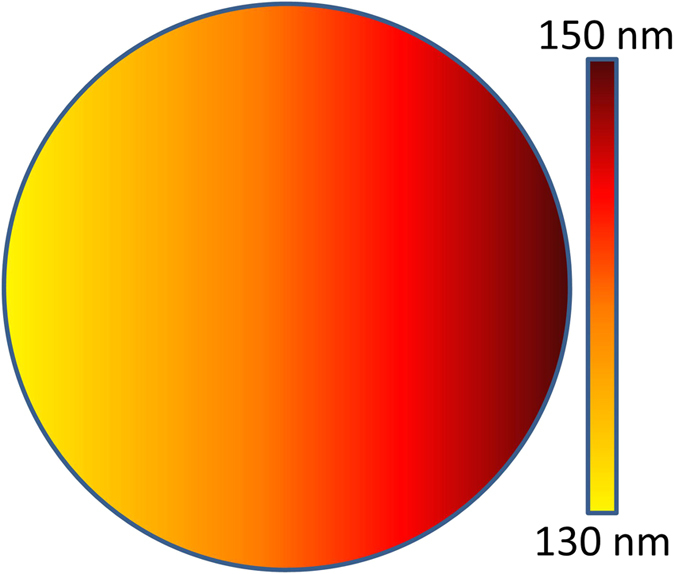
Width distribution of the nanochannels in a 4 inch diameter silicon substrate. The Cu source is on the left side of the substrate. The nanochannels become wider from left to right.

The width distribution over the entire area is illustrated in Fig. 9, where the Cu source is placed left of the substrate. The width of the nanochannels increases from left to right, as they are located farther and farther away from the Cu source.

As for the depth of the nanochannels, it also varies somewhat by their location on the wafer, probably because of non-uniform silicon etching. As shown in Table 2, the maximum depth difference is 5 nm, and at an average nanochannel depth of 47 nm, that amounts to nearly 11%. This non-uniformity of nanochannel depth, caused by the non-uniform distribution of Ar ions in the chamber, can again be seen as a limitation of this proposed technique.

### The reproducibility of the 2D nanochannels between chips

**Figure 10:**
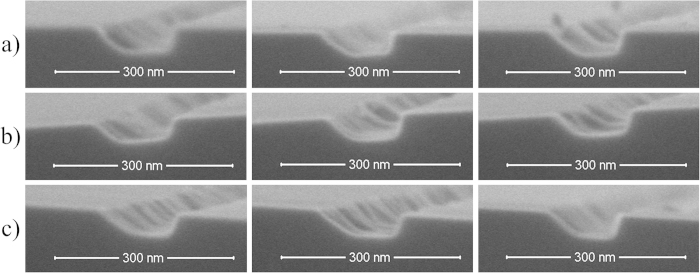
SEM images of 2D silicon nanochannels in different chips. The images (**a**) to (**c**) show the nanochannels from three silicon substrates fabricated by the proposed technique. The Cu deposition angle is 46.2° and the sputter etching time is 25 min. (**a**) 130 ± 2 nm wide and 43 ± 1 nm deep nanochannel, (**b**) 127 ± 1 nm wide and 40 ± 1 nm deep nanochannel, and (**c**) 130 ± 7 nm wide and 41 ± 3 nm deep nanochannel.

To investigate the reproducibility of the 2D nanochannels between chips, fabrication of the nanochannel mold was repeated three times. The nanochannels were fabricated under a Cu deposition angle of 46.2° and sputter etching time of 25 min. Width and depth of the nanochannels was measured in all three chips (Fig. 10). The maximum difference in nanochannel width from chip to chip is 3 nm. At the average nanochannel width of 129 nm that amounts to a width variation of 2%. The maximum difference in nanochannel depth is about 2 nm, and at the average nanochannel depth of 41 nm that amounts to a 5% variation. The ANOVA repeated measures show no significant difference in width between chips.

**Figure 11:**
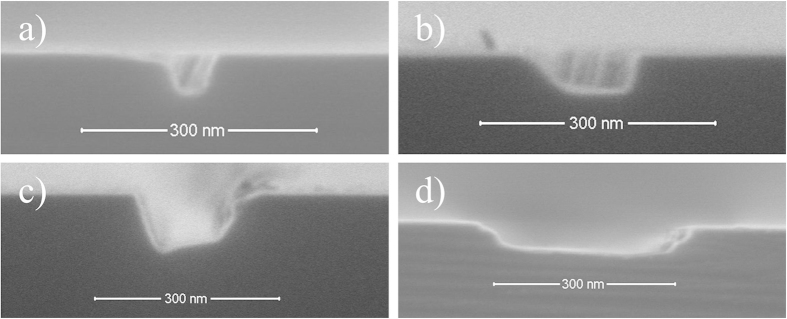
2D silicon nanochannels of different dimensions. (**a**) 57 nm wide and 52 nm deep nanochannel resulting from Cu deposition angle of 55.4° and sputter etching time of 20 min, (**b**) 138 nm wide and 47 nm deep nanochannel resulting from Cu deposition angle of 46.2° and sputter etching time of 25 min, (**c**) 160 nm wide and 87 nm deep nanochannel resulting from Cu deposition angle of 43.7° and sputter etching time of 35 min, and (**d**) 360 nm wide and 49 nm deep nanochannel resulting from Cu deposition angle of 36° and sputter etching time of 18 min.

The width of the nanochannel is determined by the Cu deposition angle, while its depth is determined by Ar ion etching time. The technique proposed in this study thus allows nanochannel fabrication in a wide range of dimensions by varying the Cu deposition angles and Ar^+^ etching time. Figure 11 shows the profiles of nano-molds with different size channels. By optimizing the Cu deposition angle and Ar^+^ etching time, silicon nanochannels can be as narrow as 57 nm and up to 52 nm deep (Fig. 11a).

### Fabrication of 2D polymer nanochannel devices using the silicon nano-mold

**Figure 12:**
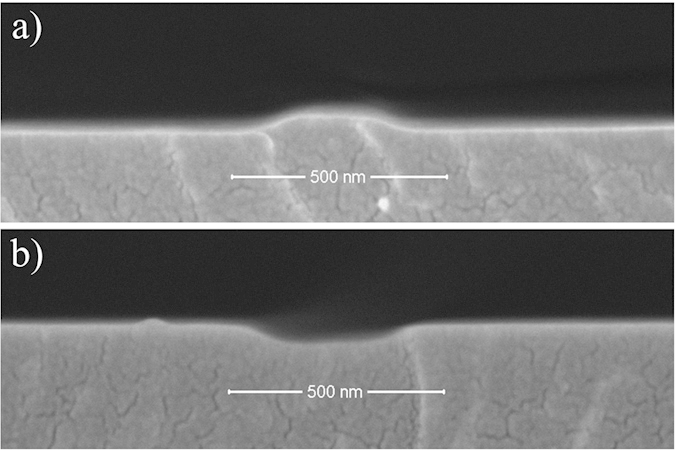
SEM images of silicon nano-mold, SU-8 nano-mold and SU-8 nanochannels. (**a**) shows the profile of a 2D SU-8 nano-ridge, 370 nm wide and 42 nm high, transferred by hot-embossing for 10 minutes at a temperature of 85 °C and pressure of 1 MPa from the concave feature of the silicon nanochannel mold. That convex nano-structure was used to heat-imprint the intended nanochannel into the final 2D SU-8 substrate chip at a temperature of 85 °C, pressure of 1 MPa, and 10 min imprinting time. (**b**) shows the profile of the final 379 wide and 40 nm deep nanochannel.

Once the technique of 2D silicon nano-mold fabrication was validated, the finished mold was used to fabricate 2D polymer nanochannel devices. As a first step the 2D silicon nano-mold was used to imprint a SU-8 nano-mold transferring the nanochannels as convex features (Fig. 11d) at an imprinting temperature of 85 °C, imprinting pressure of 1 MPa, and imprinting time of 10 min. The patterned SU-8 was then exposed to UV light using a UV mask at a dose of 227.6 μWcm^−2^ for 10 min. After UV exposure and postbake, the epoxy groups cross-linked with polyfunctional amine, then the glass transition temperature of SU-8 rose from 55 °C to 200 °C. The nano-ridges of the 2D SU-8 nano-mold were 370 nm wide and 42 nm high (Fig. 12a). After improving the anti-adhesion properties of the SU-8 nano-mold by treating it with trimethylchlorosilane, it was used to imprint its nano-pattern into a SU-8 substrate at a temperature of 85 °C, pressure of 1 MPa, and time of 10 min. The resulting 2D SU-8 nanochannels were 379 nm wide and 40 nm deep (Fig. 12b) and showed a slightly deformed pattern. It is important to choose suitable materials for thermal nanoimprinting. In the present study, two replications were required to make the nanochannels of the concave silicon nano-mold appear in the intended SU-8 nanochannel chips. An issue during thermal nanoimprinting is mold deformation. Further studies should take a closer look at imprinting parameters and materials to achieve a higher precision in the fabrication of high precision nanofluidic chips.

This study proposes a low-cost, large-surface nano-mold fabrication technique for linear nanochannels. Nanochannels of linear geometry are the ones most frequently used in nanofluidic devices^13^,^38^,^39^,^40^. They have become crucial devices with a wide variety of biological and chemical applications such as rapid analysis of elegans chemotaxis^41^, protein analysis^9^,^10^, ion separation^15^,^16^, DNA stretching and mapping^11^,^12^,^13^, as well as in energy absorption systems^17^,^18^.

However, this technique still needs to overcome some limitations. The beds of the nanochannels are not perfectly smooth. Deformations are caused by slightly uneven lines of the photolithography mask used in the present study, which caused a series of deformation in the photoresist, in the Si substrate, and thus also in the nanochannel beds. A higher accuracy photolithography mask would be highly recommended for the future studies.

Width and depth of the nanochannels so far lack uniformity. The width of the nanochannels varies depending on their location in the Si substrate. They are narrower near the Cu source than farther away from it, since inclined Cu deposition at any angle results in wider Cu nano-mask slits at a greater distance between Cu source and Cu nano-mask gap. The depth of the nanochannel is effected by the distribution of Ar ions in the chamber of the thin film deposition system during the Ar ion sputter etching step. The uniformity of the nano-channel could be improved by carefully controlling the deposition system in future studies.

## Conclusion

In the commercial production of nano-structured components high throughput is very desirable. For nanochannel technologies that can also mean chips of as large a surface as possible. In this study we present a method of fabricating linear nanochannels over a 4 inch diameter surface by inclined Cu deposition and Ar^+^ etching. The experiments conducted to assess the uniformity and accuracy of the nanochannels over the whole chip area showed variations of up to 10% in width and 11% in depth, a greater than desirable variability. However, when several chips were produced from the same mold, dimensions from chip to chip varied very little: 2% in width and 5% in depth, which shows a high degree of reproducibility. Since all the production steps could be carried out without expensive materials or equipment and because the results proved to be highly reproducible, the method proposed has considerable merit for the larger-volume production of large-surface nanofluidic chips used in applications where dimensional variations of 10% and slightly above can be tolerated. Those applications could range from liquid pumping and transport control to energy conversion and separation. It can be expected that the proposed nanostructure fabricating technique will play an important role in a number of fields, both in the production of scientific devices and in fundamental research.

## Additional Information

**How to cite this article**: Yin, Z. *et al.* A novel 2D silicon nano-mold fabrication technique for linear nanochannels over a 4 inch diameter substrate. *Sci. Rep.* **6**, 18921; doi: 10.1038/srep18921 (2016).

## Supplementary Material

Supplementary Information

## Figures and Tables

**Table 1:** Dimensional uniformity of the micro-mesas throughout the 4 inch area.

Location	Width (nm)	Height (nm)
a)	997 ± 44	414 ± 7
b)	1003 ± 38	420 ± 6
c)	984 ± 23	415 ± 10
d)	944 ± 29	418 ± 10
e)	959 ± 27	412 ± 9

**Table 2:** Uniformity of the nanochannels over the 4 inch diameter area.

Location	Width (nm)	Depth (nm)
a)	143 ± 2	48 ± 3
b)	134 ± 3	48 ± 3
c)	130 ± 2	43 ± 1
d)	144 ± 3	48 ± 4
e)	139 ± 1	47 ± 4

The Cu deposition angle is 46.2° and the sputter etching time is 25 min.
