# Supplementary material for: A novel 2D silicon nano-mold fabrication technique for linear nanochannels over a 4 inch diameter substrate
Source: Sci Rep. 2016 Jan 11;6:18921. doi: 10.1038/srep18921 (PMC4707436; doi:10.1038/srep18921)
Supplement: Supplementary Information [file srep18921-s1.doc]

Supplementary Information:

A novel 2D silicon nano-mold fabrication technique for linear nanochannels over 4 inch large area

**Zhifu Yin^1^, Liping Qi^2^, Helin Zou^1,3,*^,Lei Sun^1^**

^1^*Key Laboratory for Micro/Nano Technology and Systems of Liaoning Province, Dalian University of Technology, Dalian 116024, China*

^2^*Department of Biomedical Engineering, Dalian University of Technology, Dalian 116024, China*

^3^*Key Laboratory for Precision and Non-traditional Machining Technology of Ministry of Education, Dalian University of Technology, Dalian 116024, China*

I. The linear trend of *W(α)*

During Cu deposition, the mean free path of Cu atom
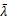
 can be expressed as^1-3^:

$\overline{\lambda}=\frac{0.667}{P}$ (1)

Where, *P* is the chamber pressure.

To avoid the collision between Cu atoms, and between Cu atoms and the gas molecules, the mean free path of Cu atom ($\overline{\lambda}$) must be ten times the distance between the Cu source and the substrate (*D*)^1, 2^,

$\overline{\lambda}\geq10D$ (2)

Where, *D* is the vertical distance between the Cu source and the substrate.

To avoid collisions, the following relationship should be met (In conjunction with the Eqs. 1 and 2),

$P\leq\frac{6.67\times10^{-2}}{D}$ (3)

In our metal film thermal deposition system (ZZS400, costar group, China), *D* is 33 cm. Consequently, when *P* is smaller than 2×10^-3^ Pa, the Cu atoms rarely collides with the gas molecules. In our deposition system, the pressure was controlled at below 0.7×10^-3^ Pa, which means that during Cu deposition collisions between Cu atoms and between Cu atoms and gas molecules were quite unlikely. The Cu atoms can nearly diffuse along a straight line. Then linear trend of *W(α)* can be explained by means of trigonometry,

As shown in Fig. S1, the deposition angle *α_1_*, *α_2_* can be expressed as,

*α_1_*=*arctan*(*D*/*L*) (4)

*α_2_*=*arctan*(*D*/(*L*+*L_1_*)) (5)

where, *α_1_*, *α_2_* are the Cu deposition angles, *D* is the vertical distance between Cu source and the substrate (In this paper *D* is 33 cm), *L* is the horizontal distance between Cu source and the substrate (In this paper *L* is 32 cm), *L_1_* is the diameter of the Si substrate (In this paper *L_1_* is 10.16 cm). So *α_1_* is 45.9°, and *α_2_* is 38.1°. It means the Cu deposition angle ranges from 38.1° to 45.9° over a 4 inch Si substrate.

According to Fig. 4, the width of the nanochannels can be evaluated by the height of the micro-mesa and the Cu deposition angle.

*W*= *H_mesa_* cot(*α*) (6)

Where, *W* is the width of the nanochannel, *H_mesa_* is the height of the micro-mesa and *α* is the Cu deposition angle.

Fig. S2 shows the curves of the cot(*α*). From 38.1° to 45.9°, cot(*α*) can be seen as a linear function. Therefore, according to Eq. (6), when the height of the micro-mesas is constant, the width of the nanochannels and the Cu deposition angle can be in have a linear relationship.


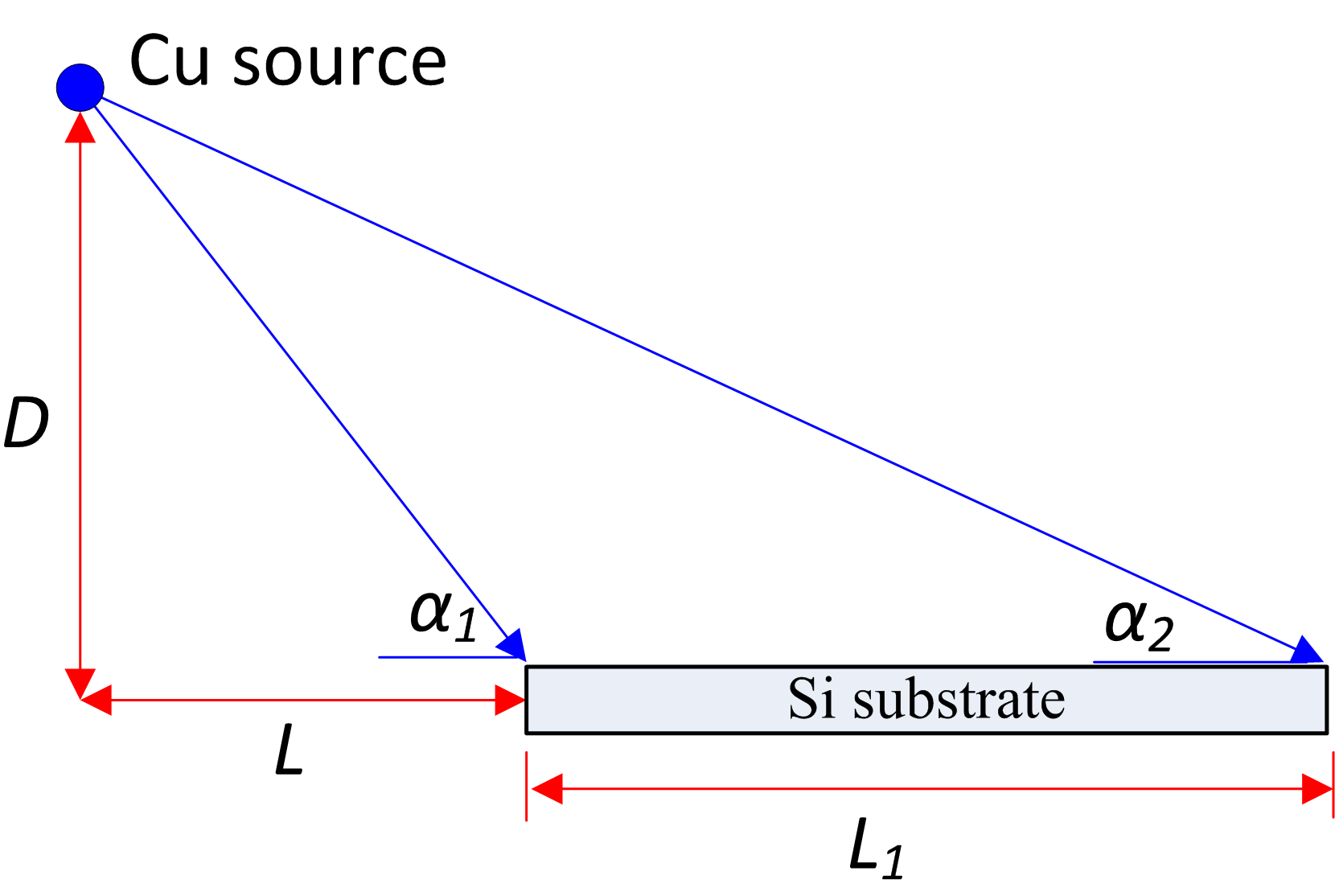


Figure S1. Range of deposition angles over 4 inch Si substrate. *D* is the vertical distance between Cu source and the substrate, *L* is the horizontal distance between Cu source and the edge of the Si substrate, *L_1_*is the diameter of the Si substrate.


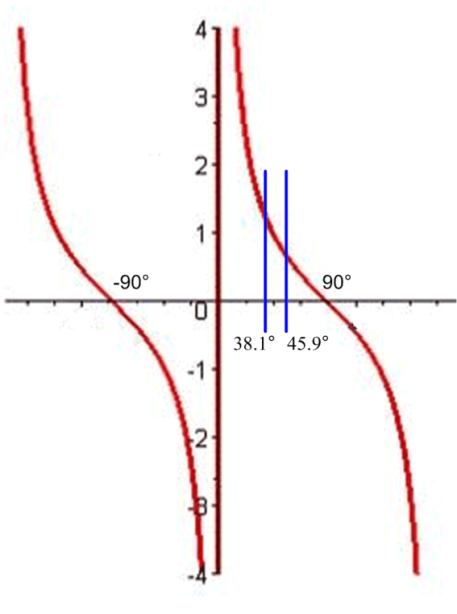


Figure S2. The curves of the cotangent function Cot(*α*) can be seen as a linear function when *α* ranges from 38.1° to 45.9°.

References:

1. Tian, M.B., *Thin film technologies and materials*. 2006, Tsinghua University Press: Beijing, China. p. 442-455.

2. Ohring, M., ed. *Materials science of thin films*. 2nd Edition ed. 2002, Academic Press: Pittsburgh, US.

3. Guan, X., ed. *Foundation of silicon integrated circuit technology*. 2003, Peking university press: Peking, China.

II. Etching-and-cooling process

The temperature issue can be solved by an etching-and-cooling process.

The steps of the new etching strategy are as follows: 1) Etching step: the silicon substrate was etched for 10 min. The temperature increased by 5°C. 2) Cooling step: the substrate was cooled down to room temperature by natural cooling (about 10 min); 3) Etching step: the silicon substrate was etched for the second time (10 min); 4) Cooling step: the substrate was cooled down to room temperature again. We repeated the etching and cooling steps, so the temperature would remain near room temperature, keeping the etching rate fairly constant.

The influence of the Ar^+^ etching time on the depth of the nanochannels by using etching-and-cooling process is shown in Fig. S3. In Fig. S3 it can be seen that there is a linear relationship between the Ar^+^ etching time and the depth of the nanochannels. The etching rate is nearly uniform. Fig. S4 shows the SEM image of the nanochannel etched by etching-and-cooling process.


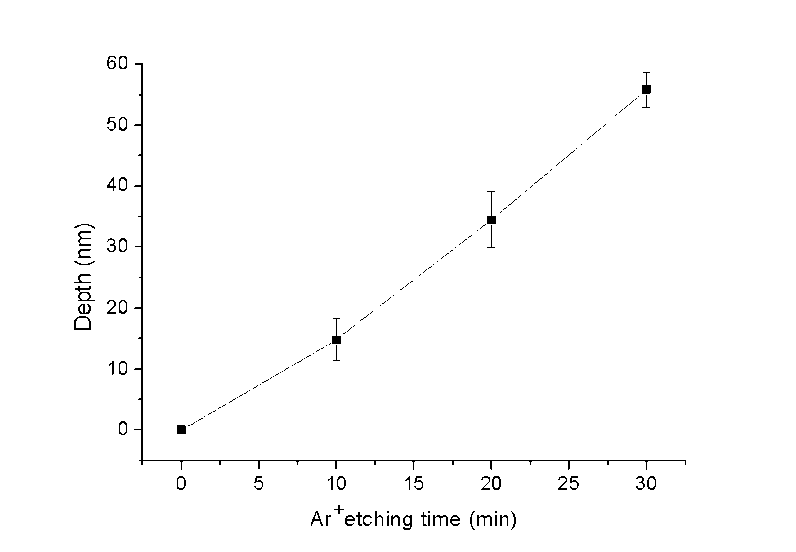


Supplementary Figure S3. The relationship between the Ar^+^ etching time and the depth of the nanochannels by using etching-and-cooling process.


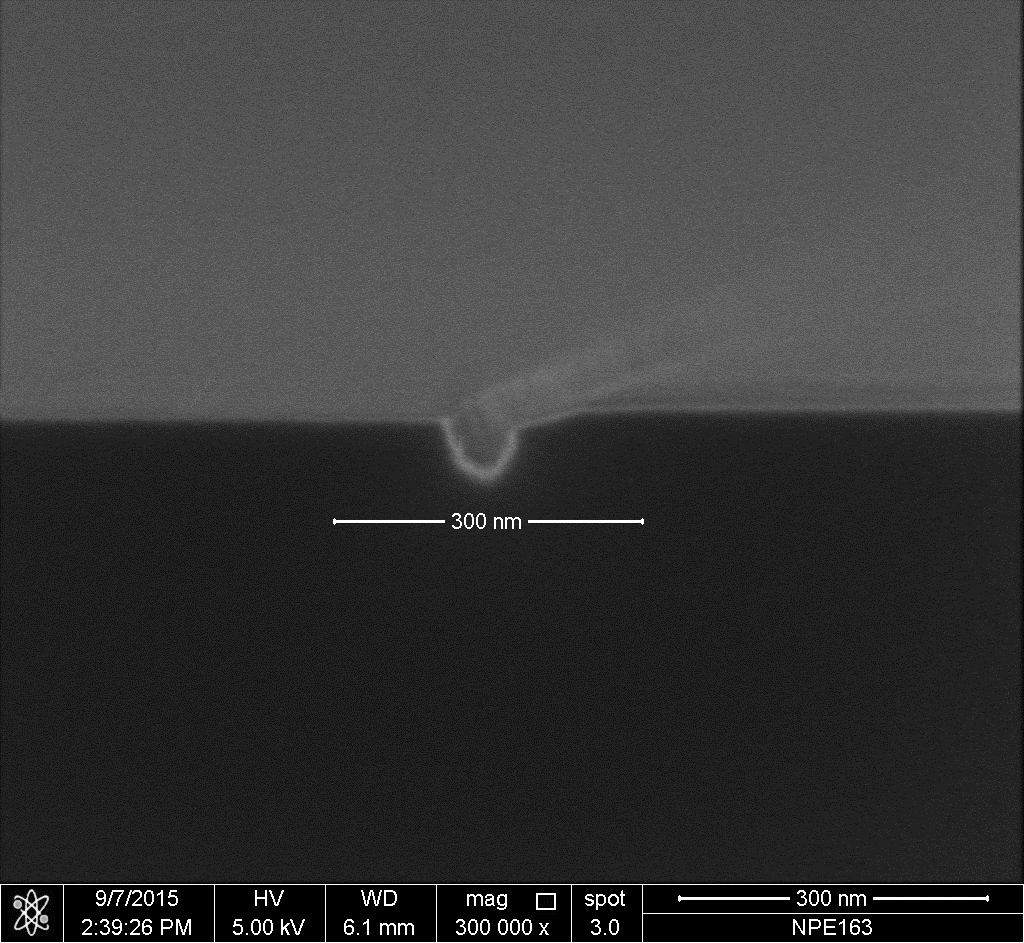


Supplementary Figure S4. The SEM image of the nanochannel etched by etching-and-cooling process. The depth of the nanochannel is 55 nm. Cu deposition angle is 55.4° and etching time is 30 min
